# C/EBPα Is Required for Long-Term Self-Renewal and Lineage Priming of Hematopoietic Stem Cells and for the Maintenance of Epigenetic Configurations in Multipotent Progenitors

**DOI:** 10.1371/journal.pgen.1004079

**Published:** 2014-01-09

**Authors:** Marie S. Hasemann, Felicia K. B. Lauridsen, Johannes Waage, Janus S. Jakobsen, Anne-Katrine Frank, Mikkel B. Schuster, Nicolas Rapin, Frederik O. Bagger, Philipp S. Hoppe, Timm Schroeder, Bo T. Porse

**Affiliations:** 1 The Finsen Laboratory, Rigshospitalet, Faculty of Health Sciences, University of Copenhagen, Copenhagen, Denmark; 2 Biotech Research and Innovation Center (BRIC), University of Copenhagen, Copenhagen, Denmark; 3 Danish Stem Cell Centre (DanStem) Faculty of Health Sciences, University of Copenhagen, Copenhagen, Denmark; 4 The Bioinformatic Centre, Department of Biology, Faculty of Natural Sciences, University of Copenhagen, Copenhagen, Denmark; 5 Department of Biosystems Science and Engineering, ETH Zurich, Basel, Switzerland; Cincinnati Children's Hospital Medical Center, United States of America

## Abstract

Transcription factors are key regulators of hematopoietic stem cells (HSCs) and act through their ability to bind DNA and impact on gene transcription. Their functions are interpreted in the complex landscape of chromatin, but current knowledge on how this is achieved is very limited. C/EBPα is an important transcriptional regulator of hematopoiesis, but its potential functions in HSCs have remained elusive. Here we report that C/EBPα serves to protect adult HSCs from apoptosis and to maintain their quiescent state. Consequently, deletion of *Cebpa* is associated with loss of self-renewal and HSC exhaustion. By combining gene expression analysis with genome-wide assessment of C/EBPα binding and epigenetic configurations, we show that C/EBPα acts to modulate the epigenetic states of genes belonging to molecular pathways important for HSC function. Moreover, our data suggest that C/EBPα acts as a priming factor at the HSC level where it actively promotes myeloid differentiation and counteracts lymphoid lineage choice. Taken together, our results show that C/EBPα is a key regulator of HSC biology, which influences the epigenetic landscape of HSCs in order to balance different cell fate options.

## Author Summary

Hematopoietic stem cells (HSCs) are required for the lifelong generation of blood cells. To fulfill this requirement HSCs carefully balance cell fate decisions such as self-renewal, differentiation, quiescence, proliferation and death. These features are regulated in part by transcription factors, which act by controlling the expression of genes important for the functional properties of HSCs. C/EBPα is a well-known inducer of myeloid differentiation. It is lowly expressed in HSCs and its potential function in these cells has been extensively debated. Here, we demonstrate that *Cebpa* deletion impacts on HSC self-renewal, differentiation, quiescence and survival. Through gene expression and ChIP-seq analyses of stem and progenitor cell-enriched cell populations, we further show that C/EBPα binds to regulatory regions of genes that are induced during granulocytic differentiation, suggesting that C/EBPα acts to prime HSCs for differentiation along the myeloid lineage. Finally, we demonstrate that C/EBPα loss leads to epigenetic changes at genes central to HSC biology, which implies that it may act to recruit chromatin writers/erasers through mechanisms that remain to be characterized. In conclusion, our work identifies C/EBPα as a central hub for HSC function and highlights how a single transcription factor may coordinate several HSC fate options.

## Introduction

Hematopoietic stem cells (HSCs) are responsible for the maintenance of a constant production of blood cells throughout life. To achieve this, HSCs have to tightly regulate their different fate options including self-renewal, proliferation, differentiation and apoptosis, as alterations in any of these may lead to HSC exhaustion, expansion or leukemia [1]. HSC fate options are controlled by a number of different pathways and are influenced both by the microenvironment and by the actions of cell-autonomous regulators such as transcription factors (TFs) and chromatin-interacting proteins [2].

Given their impact on gene expression, the influence of TFs on HSC properties has been the focus of several studies. Indeed, factors such as C-MYB, ERG, and PU.1 are all essential for preserving HSC self-renewal and their deletion have dramatic impact on hematopoietic maintenance both during fetal and adult life [3], [4], [5], [6]. Other factors, as exemplified by SOX17, are required exclusively for the maintenance of fetal HSCs, whereas GFI-1 and ETV6 only appear to play a role in an adult setting [7], [8], [9].

TF function is interpreted in a chromatin context and, accordingly, chromatin readers and writers have been shown to be important for HSC function and maintenance. Examples include the PRC1 component BMI-1 [10], [11], the maintenance DNA methyltransferase DNMT1 [12], [13] as well as the H3K4 methyltransferase MLL1 [14]. Despite the importance of both TFs and chromatin context for HSC function, our knowledge on how TF binding is interpreted within an epigenetic landscape, and how they may influence epigenetic configurations remains limited. Importantly, given their inherent developmental plasticity, stem cells have been reported to exhibit unique epigenetic signatures of which the so-called bivalent configuration is the best characterized. Work in ES cells has shown that bivalently marked genes are lowly expressed, enriched in genes involved in development/differentiation, and display active (H3K4me3) as well as repressive (H3K27me3) histone marks [15], [16]. As stem cells progress along the path of differentiation the bivalent configuration is resolved into an active or repressed state with a concomitant upregulation or downregulation, respectively, of the expression of previously marked genes [15], [16]. To what extent the bivalent signature is influenced by loss of TFs in HSCs has not been characterized.

C/EBPα is an important myeloid TF that functions not only by binding to regulatory DNA elements and directing transcription, but also through its ability to constrain proliferation by inhibiting the transcriptional activity of E2F-complexes [17], [18], [19], [20]. In the hematopoietic system loss of C/EBPα leads to a differentiation block upstream of the Granulocytic Monocytic Progenitor (GMP) accompanied by an accumulation of earlier stem and myeloid progenitor populations [17], [21].

In acute myeloid leukemia (AML), *CEBPA* is found mutated in approximately 10% of cases, and studies in mouse have shown that the tumor-suppressive functions of C/EBPα can be ascribed to its ability to balance the proliferation and differentiation of hematopoietic stem and progenitor cells (HSPCs) populations [18], [22]. Indeed, HSCs from mice harboring tumor-prone variants of C/EBPα displayed altered cell cycle kinetics, but how this impacts on HSC function was difficult to assess due to the leukemic transformation in these animals. Furthermore, complete loss of C/EBPα has been reported to endow fetal HSC-enriched populations with a minor competitive advantage in a transplantation setting [21] and C/EBPα has recently been shown to control the fetal-to-adult switch of HSCs [23]. However, at present we lack knowledge on the long-term self-renewal properties of C/EBPα in normal adult HSCs as well as insights into how it potentially may influence transcription and the epigenetic configuration of these cells.

In the present work we have used mouse genetics to characterize the role of C/EBPα in adult HSCs. We found that loss of *Cebpa* leads to HSC exhaustion, identifying C/EBPα as a crucial regulator of HSC self-renewal. To molecularly assess the function of C/EBPα in HSCs, we combined gene expression analysis with genome-wide profiling of key epigenetic marks and C/EBPα binding using Chromatin ImmunoPrecipitation coupled to sequencing (ChIP-seq). Our findings demonstrate that C/EBPα modulates key pathways associated with HSC function and differentiation, in part by affecting the epigenetic states of genes associated with some of these.

## Results

### Loss of C/EBPα Affects the Numbers of Immunophenotypic HSPCs

To investigate the functional importance of C/EBPα in the HSPC compartment, we generated *Cebpa* ^fl/fl^ and *Cebpa* ^fl/fl^;*Mx1Cre* mice (hereafter termed *Cebpa* ^fl/fl^ and *Cebpa* ^Δ/Δ^, respectively) and injected them with pIpC. Mice were analyzed 18–21 days later at which time point the *Cebpa* allele was completely recombined in *Cebpa* ^Δ/Δ^ mice (Figure S1A). Of note, bone marrows (BM) of *Cebpa* ^Δ/Δ^ mice had an approximately 30% reduction in cellularity compared to *Cebpa* ^fl/fl^ littermates due to the loss of neutrophil granulocytes (Figure S1B, C).

**Figure 1:**
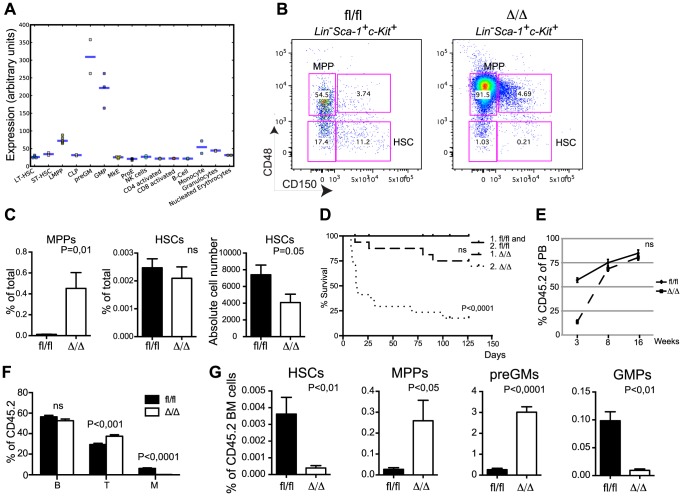
Loss of *Cebpa* affects HSC self-renewal. (A) Expression of *Cebpa* in different hematopoietic cell populations derived from the HemaExplorer website (http://servers.binf.ku.dk/hemaexplorer/). (B) HSC analysis of *Cebpa* ^fl/fl^ and *Cebpa* ^Δ/Δ^ BM. (C) Quantification of the data in (B). *Cebpa* ^fl/fl^ (n = 7) and *Cebpa* ^Δ/Δ^ (n = 7). Each panel shows one representative experiment out of three. (D) Survival curves of primary and secondary recipients transplanted with *Cebpa* ^fl/fl^ (primary: n = 12, secondary: n = 16) and *Cebpa* ^Δ/Δ^ (primary: n = 16, secondary: n = 17) BM. (E) Contribution of CD45.2 cells to the blood of primary recipients. *Cebpa* ^fl/fl^ (n = 12) and *Cebpa* ^Δ/Δ^ (n = 16). (F) Distribution of B, T and myeloid cells in the CD45.2+ blood of primary recipients 16 week after transplantation. (G) BM analysis of primary recipients of *Cebpa* ^fl/fl^ (n = 8) or *Cebpa* ^Δ/Δ^ (n = 8) BM cells. Data are represented as mean+SEM, ns designates not significant.

In line with its requirement for myeloid differentiation [21] publically available gene expression profiles (http://servers.binf.ku.dk/hemaexplorer/) [24] revealed a distinct expression pattern in which *Cebpa* is lowly expressed in the stem cell compartment and becomes transiently upregulated during commitment to the granulocytic monocytic lineage (Figure 1A). Flow cytometry-based analyses of the myelo-erythroid progenitor compartment verified the reported block in monocytic and granulocytic differentiation at the preGM to GMP transition in *Cebpa* ^Δ/Δ^ BMs and the concomitant increase in erythroid and megakaryocytic progenitors (Figure S1D, E) [17], [21]. Moving further upstream in the hematopoietic hierarchy, we found a prominent expansion of the MPP compartment (LSK, CD150−, CD48+) whereas the frequency of immunophenotypic HSCs (LSK, CD150+, CD48−) was unaltered. However, given the reduction in BM cellularity in *Cebpa* ^Δ/Δ^ mice this translates into a 50% reduction in the absolute numbers of HSCs upon loss of C/EBPα (Figure 1B, C).

### Loss of *Cebpa* Compromises HSC Activity and Leads to Their Exhaustion

The reduction of absolute HSC numbers suggests an uncharacterized function of C/EBPα in HSC biology. To test this functionally, we first transplanted BM from *Cebpa* ^Δ/Δ^ and *Cebpa* ^fl/fl^ mice non-competitively into lethally irradiated recipient mice. Whereas all mice receiving *Cebpa* ^fl/fl^ BM cells survived, 4/16 of those receiving *Cebpa* ^Δ/Δ^ BM died within 16 weeks (Figure 1D). In the remaining mice *Cebpa* ^Δ/Δ^ donor cells contributed to the peripheral blood to a similar extent as the *Cebpa* ^fl/fl^ control at 8 and 16 weeks post-transplantation, but exhibited a lower level of reconstitution at three weeks post-transplantation due to the requirement of C/EBPα for the production of the early reconstituting neutrophil granulocytes (Figure 1E). Importantly, we find the recipients to be completely devoid of donor-derived granulocytes at all time points (Figure 1F and data not shown) showing that *Cebpa* is fully recombined in the transplanted cells and that it is equally important for granulocytic differentiation in a transplantation setting. Remarkably, the frequency of *Cebpa* ^Δ/Δ^ HSCs was reduced 20-fold in the recipient BMs 16-weeks post-transplantation, whereas the effects on other HSPC subsets were similar to those observed in non-transplanted animals (Figure 1G). To assess this further, we serially transplanted BM from primary recipients and found that only 3/17 of the secondary recipients reconstituted with *Cebpa* ^Δ/Δ^ BM survived 16 weeks post-transplantation (Figure 1D).

**Figure 2:**
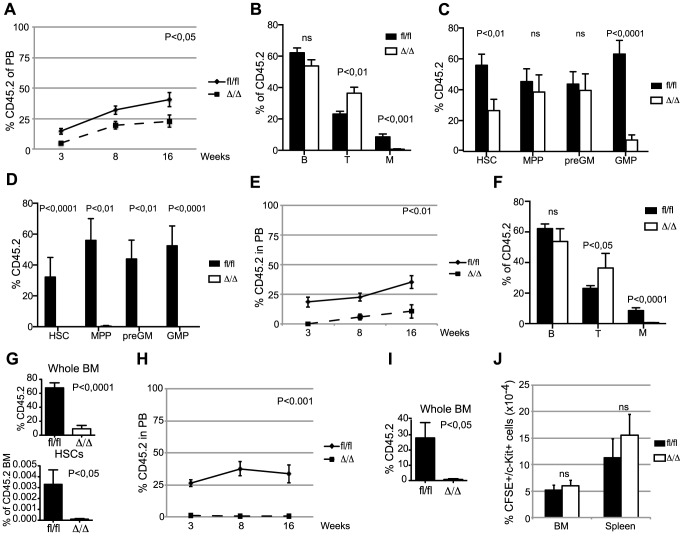
*Cebpa* ^Δ/Δ^ HSCs are functionally compromised on a cell-to-cell basis. (A) Contribution of CD45.2 cells to the blood of primary recipients. *Cebpa* ^fl/fl^ (n = 13) and *Cebpa* ^Δ/Δ^ (n = 13). (B) Distribution of B, T and myeloid cells in the CD45.2+ blood of primary recipients 16 week after transplantation. (C) BM analysis of primary recipients transplanted with *Cebpa* ^fl/fl^ (n = 11) or *Cebpa* ^Δ/Δ^ (n = 12) BM in a 1∶1 ratio with competitor BM. (D) BM analysis of secondary recipients transplanted with BM from primary recipients receiving *Cebpa* ^fl/fl^ (n = 8) or *Cebpa* ^Δ/Δ^ (n = 7) BM in a 1∶1 ratio with competitor BM. (E) Contribution of CD45.2 cells to the blood of primary recipients transplanted with either *Cebpa* ^fl/fl^ (n = 11) or *Cebpa* ^Δ/Δ^ (n = 10) HSCs. P value designates significance at 16 weeks. (F) Distribution of B, T and myeloid cells in the CD45.2+ blood of primary recipients transplanted with either *Cebpa* ^fl/fl^ or *Cebpa* ^Δ/Δ^ HSCs 16 week after transplantation. (G) Contribution of CD45.2 cells to the BM and the frequency of HSCs within the CD45.2 BM compartment in mice transplanted with either *Cebpa* ^fl/fl^ (n = 6) or *Cebpa* ^Δ/Δ^ (n = 9) HSCs (from E). (H) Contribution of CD45.2 cells to the blood of secondary recipients transplanted with BM from primary recipients receiving either *Cebpa* ^fl/fl^ (n = 6) or *Cebpa* ^Δ/Δ^ (n = 6) HSCs. P value designates significance at 16 weeks. (I) Contribution of CD45.2 cells to the BM of secondary transplanted mice. *Cebpa* ^fl/fl^ (n = 6) and *Cebpa* ^Δ/Δ^ (n = 7). (J) Homing analysis of HSCs from *Cebpa* ^Δ/Δ^ (n = 4) or *Cebpa* ^fl/fl^ (n = 6) mice. Data are represented as mean+SEM, ns designates not significant.

Competitive BM transplantation remains the gold standard for the assessment of HSC function and we therefore transplanted CD45.2 donor cells along with equal amounts of CD45.1 competitor cells. Despite the comparable HSC frequencies in *Cebpa* ^fl/fl^ and *Cebpa* ^Δ/Δ^ donors, loss of *Cebpa* leads to a marked reduction in donor contribution both to PB and to the HSC compartment of the recipients at 18-week post-transplantation (Figure 2A–C). Importantly, the competitive disadvantage of *Cebpa* ^Δ/Δ^ BM cells was further exacerbated upon re-transplantation of 1 million donor cells from primary recipients having equal donor contributions, and resulted in their complete failure to contribute to the HSPC compartment of secondary recipients (Figure 2D). Next, we transplanted 20 highly purified (LSK, CD150+, CD48−, CD34−) HSCs from *Cebpa* ^fl/fl^ or *Cebpa* ^Δ/Δ^ BM together with 200.000 unfractionated competitor BM cells. In accordance with our previous findings, *Cebpa* ^Δ/Δ^ HSCs were markedly impaired in hematopoietic reconstitution and their contribution to the recipient HSC compartment was negligible (Figure 2E–G). Furthermore, upon re-transplantation of the BM from primary recipients, *Cebpa* ^Δ/Δ^ donor cells were unable to contribute to hematopoietic reconstitution in secondary recipients (Figure 2H, I). Finally, by transplanting 3000 CFSE-labeled HSCs (LSK, CD150+, CD48−) and assessing their homing to hematopoietic organs we could exclude any obvious role of C/EBPα in HSC trafficking during hematopoietic reconstitution (Figure 2J).

In conclusion, loss of *Cebpa* has profound effects on hematopoietic reconstitution both in competitive and non-competitive settings and collectively our functional analyses demonstrate an essential role of C/EBPα in HSC self-renewal and in preventing HSC exhaustion.

### *Cebpa* Maintains HSC Quiescence and Protects Them from Cell Death

Several reports have demonstrated that C/EBPα acts to restrict proliferation and a number of distinct mechanisms have been proposed (reviewed in [25]).

**Figure 3:**
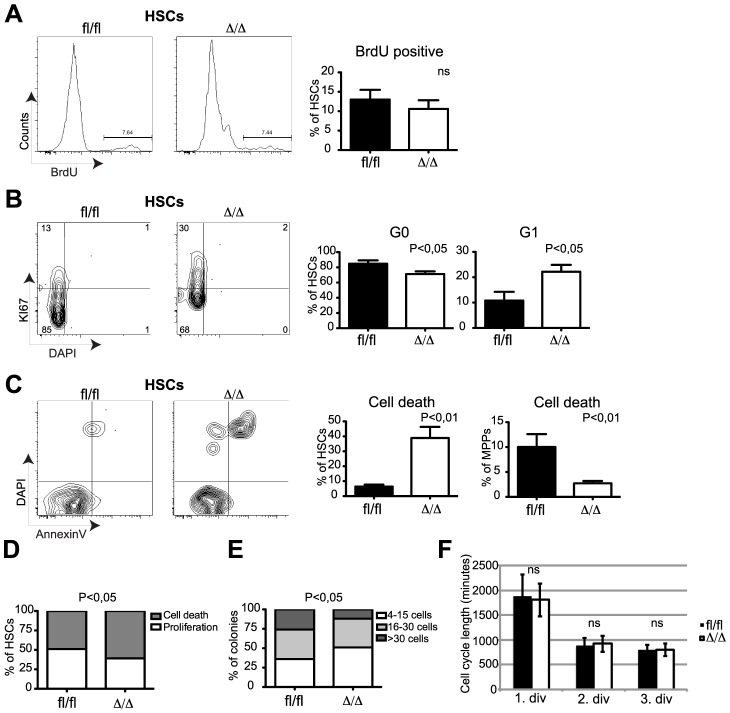
*Cebpa* ^Δ/Δ^ HSCs do not have a proliferative advantage but are prone to cell death. (A) Incorporation of BrdU into *Cebpa* ^fl/fl^ (n = 7) or *Cebpa* ^Δ/Δ^ (n = 7) HSCs. (B) Cell cycle analysis of *Cebpa* ^fl/fl^ (n = 10) or *Cebpa* ^Δ/Δ^ (n = 17) HSCs and MPPs using Ki67 and DAPI. (C) Cell death analysis of *Cebpa* ^fl/fl^ (n = 7) or *Cebpa* ^Δ/Δ^ (n = 10) HSCs using Annexin V and DAPI (D) Colony forming ability of *Cebpa* ^fl/fl^ (n = 9) or *Cebpa* ^Δ/Δ^ (n = 9) HSCs in SFEM. (E) *Cebpa* ^fl/fl^ (n = 7) or *Cebpa* ^Δ/Δ^ (n = 7) HSC colony size in SFEM. (F) Division rate of *Cebpa* ^fl/fl^ (n = 5) and *Cebpa* ^Δ/Δ^ (n = 5) HSC measured by long-term single cell tracking. Data are represented as mean+SEM.

To test to what extent the anti-proliferative properties of C/EBPα may explain the loss of self-renewal in *Cebpa* deficient HSCs, we first analyzed the frequencies of replicating HSCs by *in vivo* BrdU incorporation. Surprisingly, we were unable to detect any major differences in the amounts of BrdU+ HSCs within *Cebpa* ^fl/fl^ and *Cebpa* ^Δ/Δ^ BMs (Figure 3A), suggesting that C/EBPα does not affect DNA synthesis in HSCs *per se*. In contrast, we found that loss of *Cebpa* lead to a marked exit from quiescence as evidenced by a reduction of *Cebpa* ^Δ/Δ^ HSCs in G0 (Ki67 negative) and a compensatory increase of *Cebpa* ^Δ/Δ^ HSCs in G1 (Figure 3B). Finally, we also found that the exit from quiescence was accompanied by a marked increase in *Cebpa* ^Δ/Δ^ HSCs that underwent cell death and that this effect was restricted to immunophenotypic HSCs (Figure 3C).

We next tested the behavior of HSCs *in vitro* by first evaluating proliferation and cell death of single-sorted HSCs. Consistent with our *in vivo* analysis we found that loss of *Cebpa* resulted in both fewer and smaller colonies accompanied by an increase in cell death (Figure 3D, E). Secondly, we used single-cell tracking of HSCs *in vitro* to monitor the cell cycle length during the first three cell divisions [26]. Consistently, we detected no differences between the average cell cycle lengths by comparing *Cebpa* ^fl/fl^ and *Cebpa* ^Δ/Δ^ HSCs (Figure 3F). Thus both our *in vivo* and *in vitro* analysis demonstrated that C/EBPα does not regulate HSC proliferation *per se* but rather acts to maintain HSC quiescence and to protect HSCs from cell death.

Collectively, our phenotypic profiling of *Cebpa* deficient HSCs demonstrates that C/EBPα is a crucial regulator of the balance between quiescence, proliferation and cell death in HSCs. Hence, loss of C/EBPα leads to cell cycle entry, loss of HSC self-renewal and cell death.

### Loss of *Cebpa* Affects Gene Expression Programs Associated with Stem Cell Functions and Differentiation

**Figure 4:**
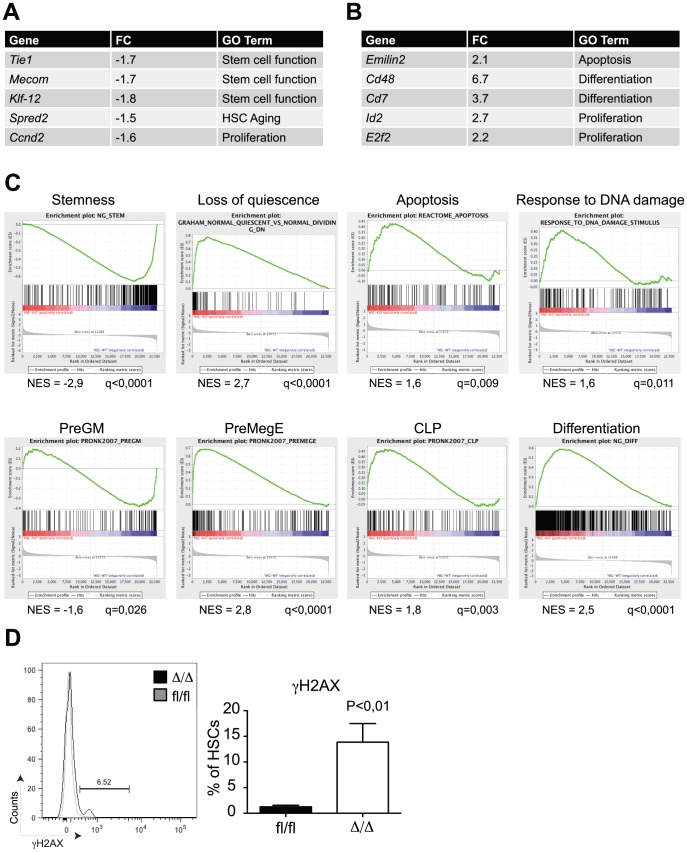
Gene expression profiling of *Cebpa* ^Δ/Δ^ HSCs shows loss of stemness and biased lineage differentiation. (A) HSCs from *Cebpa* ^Δ/Δ^ and *Cebpa* ^fl/fl^ mice were subjected to gene expression analysis. The table shows selected genes downregulated in *Cebpa* ^Δ/Δ^ compared to *Cebpa* ^fl/fl^ HSCs. (B) Selected genes upregulated in *Cebpa* ^Δ/Δ^ compared to *Cebpa* ^fl/fl^ HSCs. (C) Gene set enrichment analysis of *Cebpa* ^Δ/Δ^ HSCs compared to *Cebpa* ^fl/fl^ HSCs. (D) DNA damage as assessed by γH2AX staining of *Cebpa* ^Δ/Δ^ (n = 9) and *Cebpa* ^fl/fl^ (n = 8) HSCs. Data are represented as mean+SEM.

In order to get insights into the molecular mechanisms by which C/EBPα controls HSC self-renewal, we performed microarray-based gene expression analysis of *Cebpa* ^fl/fl^ and *Cebpa* ^Δ/Δ^ HSCs (LSK, CD150+, CD48−). In total, we found 110 and 261 genes to be more than 1.5x down- and upregulated, respectively, in *Cebpa* ^Δ/Δ^ HSCs (P<0,05) (Figure 4A, B, , Figure S2A for verification by qRT-PCR). Consistent with our phenotypic analysis, gene set enrichment analysis (GSEA) demonstrated a marked negative correlation between HSC signature genes and the *Cebpa* ^Δ/Δ^ genotype (Figure 4C). Among the gene sets correlating positively with the *Cebpa* ^Δ/Δ^ genotype we found numerous signatures involved in quiescence/proliferation, which are likely to underlie the loss of quiescence that we observed in *Cebpa* ^Δ/Δ^ HSCs. Similarly, we also found a positive correlation between the expression of apoptotic signatures and the *Cebpa* ^Δ/Δ^ genotype as well as an up-regulation of gene sets involved in the DNA damage response pathway. The relevance of the latter is supported by the 10-fold increase of γH2AX (a marker of DNA damage) positive HSCs that we observe in *Cebpa* ^Δ/Δ^ BMs (Figure 4D). Thus the increased levels in cell death and DNA damage observed upon loss of *Cebpa* are supported both by phenotypic and gene expression analyses.

Finally, we noted that a number of gene signatures representing distinct hematopoietic lineages were selectively deregulated in *Cebpa* ^Δ/Δ^ HSCs. Thus whereas the expression of granulocytic-monocytic gene sets were downregulated, their lymphoid and erythroid counterparts correlated positively with the *Cebpa* ^Δ/Δ^ genotype. These findings strongly suggest that C/EBPα primes HSCs towards the granulocytic-monocytic lineage at the expense of lymphoid and erythroid differentiation and therefore plays a key role in specifying the lineage output from HSCs (Figure 4C).

### Identification of C/EBPα Binding Sites in HSPCs

The gene expression analysis reported above identified numerous genes that were de-regulated upon loss of C/EBPα, however whether these are direct or indirect targets remained to be determined. To assess this we performed ChIP-seq analysis for C/EBPα binding in LSK cells, which contains a mixture of HSCs and, predominantly, MPPs.

**Figure 5:**
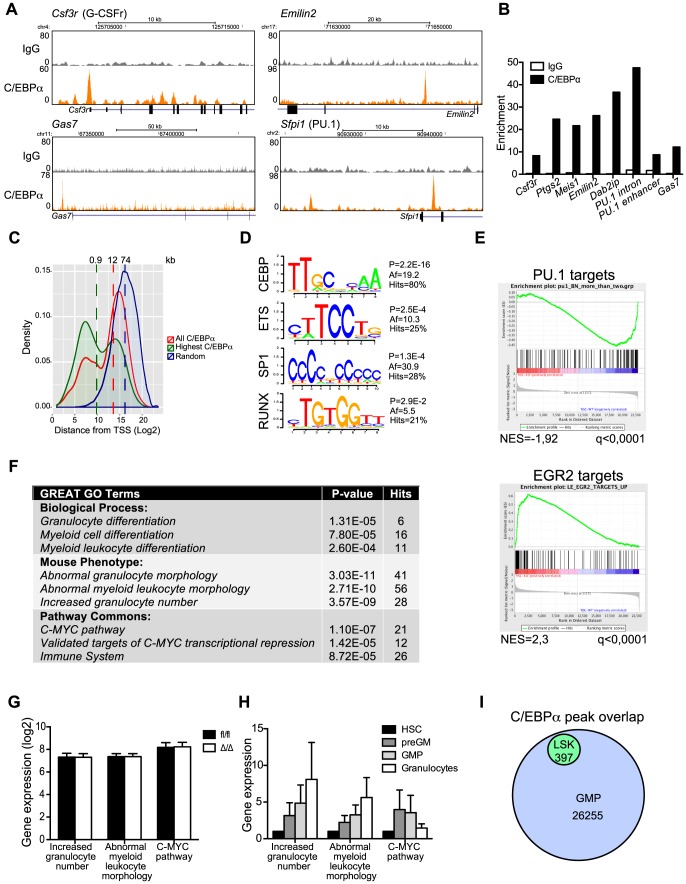
ChIP-seq analysis of C/EBPα binding in LSK cells. (A) Selected examples of ChIP-seq coverage of C/EBPα peaks in LSK cells. (B) ChIP-qPCR for IgG and C/EBPα. Enrichment was determined based on a positive vs. negative primer pair. (C) The distance from all C/EBPα peaks, the 25% highest C/EBPα peaks, or a random set, to the nearest TSS. The dashed lines represent the median. (D) Enriched TF binding motifs at C/EBPα-bound regions. P: P-value, Af: affinity score. See also Table S3. (E) Gene set enrichment analysis of *Cebpa* ^Δ/Δ^ HSCs compared to *Cebpa* ^fl/fl^ HSCs for PU.1 and EGR2 target genes. (F) GREAT analysis of C/EBPα-bound regions. See also Table S4. (G) Expression of C/EBPα targeted GO categories in *Cebpa* ^Δ/Δ^ and *Cebpa* ^fl/fl^ HSCs. (H) Relative expression of C/EBPα targeted GO categories during normal myeloid differentiation. (I) Overlap of C/EBPα bound regions in LSKs and GMPs.

Despite its low expression, we identified 397 high-confidence C/EBPα bound regions of which selected examples of ChIP-seq coverage are shown in Figure 5A (replicate 1) and verified by qPCR (Figure 5B, Table S2 for the full dataset and Figure S2B–D for data concerning the second replicate). In general, the C/EBPα bound regions were located closer to the transcription start site (TSS) of the nearest gene than a random set of regions, and 39% were located at proximal promoters within 5 kb from TSS (Figure 5C, ). Gratifyingly, motif search analysis in the immediate vicinity of the C/EBPα peak summit (+/−70 bps) identified C/EBP consensus motifs to be markedly overrepresented, however we also identified enrichment of RUNX, ETS and SP1 binding motifs (Figure 5D, Table S3). Interestingly, we find that PU.1 (an ETS factor) and EGR2 (reported to bind SP1 motifs [27], [28]) target genes to be downregulated and upregulated, respectively in *Cebpa* ^Δ/Δ^ HSCs (LSK, CD150+, CD48−) (Figure 5E). This might suggest that C/EBPα activate and repress the expression of selected PU.1 and EGR2 target genes, respectively, in HSCs in order to restrict lymphoid and promote myeloid differentiation, thereby providing further support for its lineage priming function.

We next took advantage of the Genomic Regions Enrichment of Annotations Tool (GREAT), which predicts functions of *cis*-regulatory regions by associating proximal and distal genomic regions with putative targets [29]. Interestingly, C/EBPα associated genes were enriched in a number of gene ontology (GO) categories, the majority of which were involved in *myeloid differentiation* (Figure 5F, Table S4). Surprisingly, the expression of genes belonging to these GO categories was not prominently de-regulated in *Cebpa* ^Δ/Δ^ HSCs (Figure 5G). One explanation could be that C/EBPα marks genes, which are to be expressed at latter stages of myeloid differentiation. Indeed, we find that genes belonging to the GO categories *abnormal myeloid leukocyte morphology* and *increased granulocyte numbers*, which were enriched for C/EBPα-associated genes, were upregulated during myeloid differentiation (Figure 5H). Finally, when we compare the overlap between C/EBPα bound regions in LSK cells to a similar dataset derived from GMPs, we find all regions bound by C/EBPα in LSKs to be occupied in GMPs as well. (Figure 5I, Table S5). Thus our data is consistent with a lineage priming function for C/EBPα.

Finally, in order to identify direct C/EBPα target genes in HSCs, we correlated changes in gene expression in *Cebpa* ^Δ/Δ^ HSCs with the nearest C/EBPα bound region identified by ChIP-seq in LSK cells. In total 6/102 genes (6%) and 21/257 (8%) of the down- and upregulated genes, respectively, contained a C/EBPα bound region within 100 kb from the TSS (Table S6). Hence, the vast majority of genes displaying altered expression in *Cebpa* ^Δ/Δ^ HSCs did not have a nearby C/EBPα binding site and are therefore unlikely to be directly regulated by C/EBPα in HSCs. The identified list of genes targeted by C/EBPα in HSCs/MPPs includes C/EBPα target genes known from other cell types such as *Hmox1* and *Itgal*, but also new and interesting target genes such as *Hmbs, Ccnd2 and Emilin2*. The latter is part of a family of extracellular matrix glycoproteins and has previously been shown to induce cell death by interacting with TRAIL receptors [30], which may explain the increased cell death that we observe in *Cebpa* ^Δ/Δ^ HSCs.

In conclusion we have identified 397 high-confidence regions bound by C/EBPα in LSK cells. C/EBPα binds to the proximal promoter and distal regulatory regions of genes involved primarily in myeloid differentiation as well as genes targeted by TFs with roles in HSC function and lymphoid differentiation. These findings suggest a role for C/EBPα in regulating lineage decisions in HSCs/MPPs.

### Loss of *Cebpa* Affects the Epigenetic Configuration of Bivalent Genes and Pathways Involved in HSC Function

**Figure 6:**
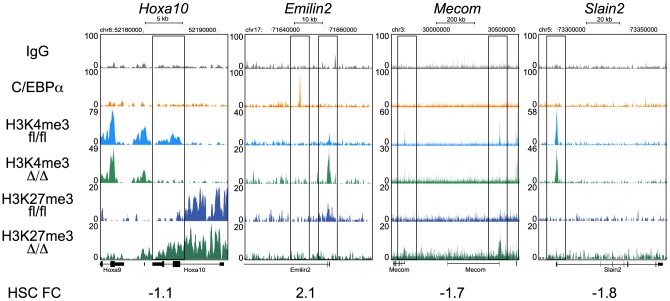
ChIP-seq analysis of H3K4me3 and H3K27me3 in *Cebpa* ^Δ/Δ^ and *Cebpa* ^fl/fl^ HSPCs. Selected examples of H3K4me3 and H3K27me3 coverage in *Cebpa* ^Δ/Δ^ and *Cebpa* ^fl/fl^ HSPCs.

Transcriptional regulation is a complex process not only involving TFs but also the chromatin context in which their function is exerted. Chromatin structure and accessibility is influenced by post-transcriptional modifications on histone tails, which are dynamically deposited and removed by chromatin modifying enzymes and serves as recruitment platforms for other chromatin binding proteins [31], [32]. To test if loss of *Cebpa* affected chromatin structure we performed replicate ChIP-seq analysis for the presence of two key histone marks, H3K4me3 and H3K27me3 using *Cebpa* ^Δ/Δ^ and *Cebpa* ^fl/fl^ LSK cells. ChIP-seq profiles of selected genes are depicted in Figure 6 (See Figure S3A–C for replicate data).

**Figure 7:**
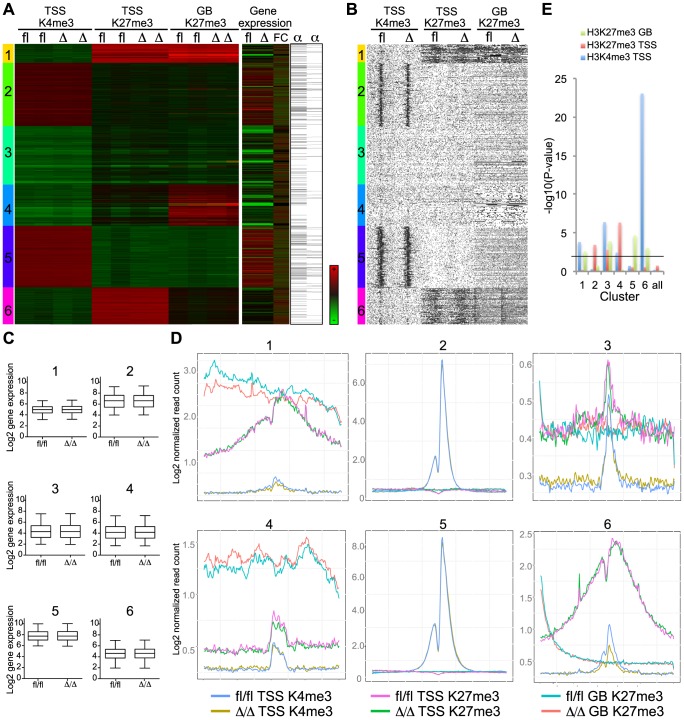
Bivalent genes have lower H3K4me3 level in *Cebpa* ^Δ/Δ^ HSPCs. (A) Clustering analysis of the H3K4me3 data at TSS and the H3K27me3 data at TSS and gene bodies. Gene expression of *Cebpa* ^Δ/Δ^ and *Cebpa* ^fl/fl^ HSCs (FC = Fold change) and C/EBPα-binding within 100.000 kb from TSS (column 1) and within 100.000 kb to nearest gene (column 2) are included. (B) Coverage density plot of the data in (A). (C) The gene expression in HSCs (log2, arbitrary units) of clusters 1–6 was investigated. (D) The coverage sums of the clusters in (A) in 10 kb windows centered on the TSS and across the gene body is shown. In cluster 2 and 5, the yellow, green and red curves are covered by the blue, pink and turquoise curves, respectively. (E) P-values of the difference in H3K4me3 in TSS, H3K27me3 in TSS and GB of clusters identified in (A). Black line indicates P = 0.01.

To systematically analyze these data, we used hierarchical clustering to separate the epigenetic profiles into distinct clusters and subsequently combined these with gene expression and C/EBPα binding data (Figure 7A). From this analysis it became evident that the vast majority of genes were highly similar in terms of their expression and H3K4me3/H3K27me3 modification patterns when *Cebpa* ^Δ/Δ^ and *Cebpa* ^fl/fl^ LSK cells were compared (Figure 7A–C). Although some changes in histone modification between the *Cebpa* ^Δ/Δ^ and *Cebpa* ^fl/fl^ genotypes reached statistical significance, they were overall small and are therefore unlikely to have major phenotypic consequences. One exception was the lowly expressed cluster 6 carrying the bivalent chromatin configuration (originally identified in ES cells [33]), which is characterized by intermediate H3K4me3/high H3K27me3 at the TSS and low H3K27me3 in the gene body. This cluster displayed a specific downregulation of the H3K4me3 mark (p = 8.93×10^−24^) around the TSS in *Cebpa* ^Δ/Δ^ LSKs (Figure 7D, E, Table S7) and similar to previous studies in ES cells, GO-analysis identified pathways involved in cell differentiation, regulation of cell differentiation and cell development to be enriched among the group of bivalently marked genes (Figure S4, Table S8). Strikingly, C/EBPα-bound regions were not over-represented in the bivalent cluster and the majority of genes in this cluster did not have a C/EBPα bound region within 100 kb of their TSS neither in the LSKs (Table S7) nor in the GMPs (data not shown). Thus, whereas C/EBPα is dispensable for the maintenance of the overall H3K4me3 and H3K27me3 modification patterns, it appears to affect the H3K4me3 status of the bivalent cluster through indirect mechanisms. This may in turn interfere with their expression later in differentiation, or alternatively, interfere with the ability of *Cebpa* ^Δ/Δ^ HSPCs to initiate differentiation.

**Figure 8:**
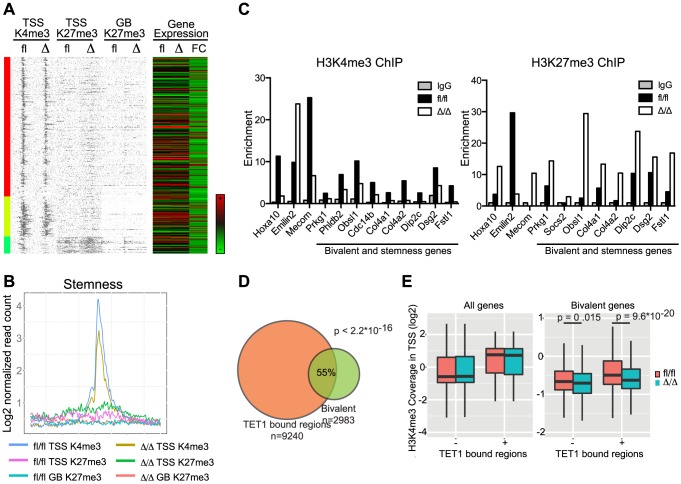
Correlation of gene expression signatures and epigenetic alterations in *Cebpa* ^Δ/Δ^ HSPCs. (A) Stemness genes were investigated for histone modifications. Coverage density plot of H3K4me3 at TSS, H3K27me3 at TSS and Gene Body of stemness genes in *Cebpa* ^Δ/Δ^ compared to *Cebpa* ^fl/fl^ LSK cells. Gene expression of stemness genes in *Cebpa* ^Δ/Δ^ compared to *Cebpa* ^fl/fl^ HSCs was included. (B) Coverage sums of the data in (A) in 10 kb windows centered on the TSS and across the gene body. (C) ChIP-qPCR for IgG, H3K4me3 and H3K27me3 on selected genes (examples from Figure 6) and genes which are part of both the bivalent cluster 6 (Figure 7A) and the stemness signature (A). For visualization purposes the H3K27me3 enrichment was normalized to IgG. (D) Overlap of TET1 bound regions in ES cells with bivalently marked genes in LSK cells. (E) H3K4me3 level in TSS of all or bivalent marked genes in *Cebpa* ^Δ/Δ^ and *Cebpa* ^fl/fl^ LSK cells.

We next took a pathway-centric approach and assessed the epigenetic changes in a selection of the gene sets identified in the GSEA analysis described above. Interestingly, for the stemness signature we observed marked changes in histone modifications in the regions flanking the TSS (Figure 8A, B). Specifically, we found the H3K4me3 (p = 1.83×10^−5^) and H3K27me3 (p = 1.41×10^−5^) marks to be reduced and increased, respectively, in *Cebpa* ^Δ/Δ^ LSK cells consistent with the changes in gene expression in HSCs. The alterations in H3K4me3 and H3K27me3 identified by ChIP-seq were verified by qPCR on selected genes as well as genes, which are part of the bivalent cluster and/or the stemness signature (Figure 8C and Figure S3D). Similar to our findings for the bivalent cluster, the majority of the genes constituting the stemness signature did not have a C/EBPα bound region within 100 kb of their TSS (Table S9), again suggesting that C/EBPα most likely affects the epigenetic marks through indirect mechanisms. For the remaining signatures we do see minor changes but none of them reached statistical significance (Figure S5).

We hypothesized that the reduced level of H3K4me3 in *Cebpa* ^Δ/Δ^ LSK cells could, in part, be explained by altered levels of H3K4me3 readers or writers. To test this we first scrutinized the HSC gene expression data for changes in the expression of candidate genes and noted that three probesets for *Tet1* were marginally downregulated (1.4-fold in all three probesets) in *Cebpa* ^Δ/Δ^ HSCs (Table S1). TET1 belongs to the TET family of 5′-methylcytosine hydroxylases, which have emerged as important epigenetic regulators of both normal and malignant hematopoiesis [34], [35], [36]. TET1 has been shown to bind CpG-islands of TSSs marked either by H3K4me3 or the bivalent H3K4me3/H3K27me3 signature in ES cells [37],[38]. To extend these data to a hematopoietic setting we used a published TET1 dataset [37] and found that genes bound by TET1 in ES cells were enriched in the group of bivalently marked genes (cluster 6) in LSK cells (Figure 8D; p<2.2×10^−16^). Moreover, when TET1 occupancy was used to stratify the bivalent genes, the TET1 bound fraction (56%) showed a marked decrease in H3K4me3 levels in *Cebpa* ^Δ/Δ^ LSKs (Figure 8E; p = 9.6×10^−20^). Interestingly, this effect was restricted to the bivalent genes as *Cebpa* ^Δ/Δ^ and control LSKs showed similar H3K4me3 levels irrespectively of the binding of TET1 (Figure 8E).

Collectively, whereas C/EBPα is largely dispensable for H3K4me3 and H3K27me3 modification patterns in HSC/MPP, its loss do lead to specific changes of the epigenetic states of bivalently marked genes as well as genes associated with stem cell function.

## Discussion

Proper control of gene transcription is crucial for essentially all biological processes and is orchestrated through a complex interplay between TFs, chromatin structure and epigenetic regulators. This also extends to the complex biology of HSCs as evidenced by the fact that mice deficient for TFs or epigenetic regulators are frequently associated with HSC phenotypes [2]. However, the mechanisms by which TFs act in a chromatin context to regulate transcription and facilitate epigenetic changes of HSCs are largely unknown.

In the present work we therefore analyzed the impact of deleting C/EBPα, one of the key transcriptional regulators of hematopoiesis, on HSC function, gene expression and the epigenetic status of key histone modifications. We find that conditional acute deletion of *Cebpa* leads to a 2-fold reduction of immunophenotypic HSCs and that *Cebpa* deficient HSCs were severely compromised. Furthermore, loss of C/EBPα promoted their exit from quiescence and was associated with a marked increase in cell death. These findings were supported by gene expression analyses, which revealed prominent transcriptional downregulation of genes associated with HSC function and quiescence as well as upregulation of genes associated with DNA damage and apoptosis. Among the pathways that displayed transcriptional changes upon loss of *Cebpa*, we find noticeable changes in the H3K4me3 and H3K27me3 marks flanking the TSS for genes involved in stem cell function (stemness signature). These genes are expressed at intermediate level (data not shown) and their epigenetic changes may reflect a drift away from an epigenetic state favoring self-renewal. In addition, whereas the overall distribution of H3K4me3 and H3K27me3 marks are largely unaltered in C/EBPα-deficient LSKs, we find that loss of C/EBPα specifically affects the H3K4me3 status of bivalent genes. Interestingly, TET1 was marginally downregulated in *Cebpa* ^Δ/Δ^ HSCs and the H3K4me3 levels were selectively reduced in *Cebpa* ^Δ/Δ^ LSKs at bivalent genes bound by TET1. Recently it was suggested that TET1, together with the epigenetic regulators CFP1 (a recruiter of the H3K4me3 methylase SETD1) and KDM2A (a H3K36 demethylase), work synergistically to maintain a distinct epigenetic configuration on their target genes [39]. Thus in this context, our findings suggest that downregulation of TET1 following loss of *Cebpa* may affect H3K4me3 status on selected genes. Whether C/EBPα affects other histone modifications such as H3K4me1 or H3K9Ac remains to be elucidated.

The importance of C/EBPα for HSC function has recently been analyzed in an adult setting. Specifically, Ye et al. [23] found that C/EBPα acts to restrict the numbers of HSCs by inhibiting their proliferation, however the effects on HSC self-renewal was not tested in a serial transplantation set-up. Furthermore, they found *N-Myc* to be bound by C/EBPα in c-Kit+ cells and suggested that loss of C/EBPα de-repressed *N-Myc* and promoted HSC expansion through an *N-Myc*-driven increase in proliferation. These findings contrast those reported in the present study, where we find C/EBPα to be important for the maintenance of a functional HSC pool as defined by serial transplantation and quantification of immunophenotypic HSCs. Moreover, in our HSC gene expression analysis, we do not find *N-Myc* to be upregulated, and C/EBPα does not bind the *N-Myc* promoter before the GMP state (data not shown), suggesting that C/EBPα regulates *N-Myc* in progenitors rather than in HSCs.

A number of methodological differences are likely to explain at least some of the differences between the findings in our study and those reported by Ye et al. First of all, whereas Ye et al. performed their analyses shortly (4–7 days after the last pIpC injection), we waited until 14–17 days after the last pIpC injection to avoid any confounding issues with the pIpC induced interferon response. Indeed, it has previously been reported that interferons influence key HSC parameters such as proliferation, apoptosis and self-renewal [40], [41], and therefore, it is likely that the HSC compartment is affected differentially 4 and 14 days after treatment with pIpC. We therefore speculate that the effect of C/EBPα loss on HSCs might evolve over time, from an initial expanding phase to a strongly compromised state ultimately resulting in increased cell death and loss of self-renewal. This would explain the increased proliferation and expansion observed early after deletion of *Cebpa* [23], the balanced HSC numbers three weeks after deletion, the loss of HSCs in primary recipients transplanted with C/EBPα-deficient HSCs/BM and the loss of re-population in secondary recipients.. It is recognized that HSC phenotypes can progress over time from proliferative to exhaustion especially under stress such as serial transplantations as shown for PTEN-deficiency [42], [43]. Moreover, although both studies used a conditional model that facilitates the full ablation of *Cebpa*, the two models are not completely identical, which could potentially explain some of the diverging results.

The functional consequences of *Cebpa* loss have also been assessed in other developmental settings. Thus, in a pre-leukemic setting, mutation of *Cebpa* leads to increased HSC proliferation but its impact on HSC function was obscured by transformation to AML [22] and in a fetal setting, E.14.5 LSK cells were reported to have a competitive advantage although their proliferative status was unaltered [21]. Together with our demonstration that loss of *Cebpa* has dramatic consequences for HSC self-renewal, these observations suggest that different mechanisms are governing HSC self-renewal during embryonic development, in the adult, and in a disease context. While this concept is not novel, and is supported by the selective importance of transcriptional regulators such as SOX17 [7] and BMI-1 [11] in fetal versus adult HSCs, C/EBPα is to our knowledge the first factor that impact oppositely on fetal and adult HSCs as well as being de-regulated in disease. Thus, our work highlights a key role of C/EBPα in HSC biology in different developmental contexts.

TFs often operate in combinations and our data suggest that at least some of the functional deficits of *Cebpa* ^Δ/Δ^ HSCs may be explained through crosstalk between C/EBPα and PU.1 - two TFs, which have been reported to act both synergistically and antagonistically in progenitor cells [44], [45]. Our findings that ETS binding sites are overrepresented in C/EBPα bound regions in LSK cells and that PU.1 target genes are downregulated in *Cebpa* ^Δ/Δ^ HSCs suggest that C/EBPα may collaborate with PU.1 to activate a transcriptional program already in HSCs. PU.1 is crucial for the development of almost all blood cells and even minor alterations in its expression level have been found to impair HSC self-renewal and lead to HSC exhaustion [46]. In this context it is tempting to speculate that C/EBPα regulates HSC self-renewal through crosstalk with PU.1 and that loss of C/EBPα reduces the output of PU.1 target genes, ultimately resulting in exhaustion of the stem cell pool.

In order to maintain hematopoietic homeostasis, HSCs have to carefully balance the choice between a number of cell fate options. Whereas early reports suggested that HSCs sampled different lineage-affiliated transcriptional programs uniformly, more recent data suggest a considerable degree of heterogeneity within the HSC compartment [47], [48], [49]. Moreover, data from cell line experiments suggest that stochastic upregulation of key regulatory molecules including lineage-instructive TFs drives differentiation along their respective lineages [50]. However, due to the technical difficulties in assessing TF binding in low-abundant cell types it is not clear whether these factors are occupying their targets already in the HSC compartment.

When analyzing the C/EBPα bound regions in the HSC/MPP-enriched LSK population we find these to be enriched for genes that were upregulated during granulocytic-monocytic differentiation. In addition, gene sets associated with myeloid differentiation are downregulated in *Cebpa* ^Δ/Δ^ HSCs, suggesting that the sampling of myeloid transcriptional programs is in part controlled by C/EBPα in HSCs perhaps involving PU.1 crosstalk. Hence, in addition to its accepted role as a lineage-instructive factor required for the transition from preGMs to GMPs [51], our data demonstrate an additional role of C/EBPα in controlling the sampling of myeloid gene programs already in HSCs through its binding to the regulatory regions of myeloid genes.

Lineage choice may be viewed as a competition between distinct lineage-affiliated programs. Interestingly, we find that loss of *Cebpa* leads to upregulation of lymphoid gene expression programs in *Cebpa* ^Δ/Δ^ HSCs, which suggests that C/EBPα may actively repress lymphoid differentiation perhaps through the inhibition of the transcriptional activities of EGR family members [52], [53]. This model is also compatible with the ability of C/EBPα to transdifferentiate lymphoid cell types into macrophages [54], [55], with the observation that a subclass of leukemia patients with silenced C/EBPα expression develops AML associated with distinct T-cell characteristics [56] and with the finding that pre-leukemic *Cebpa* mutant GMPs readily differentiate into T-cells [57].

In conclusion, we find that C/EBPα regulates HSC functions at several distinct levels. Not only is its loss associated with HSC exhaustion, but C/EBPα also appear to be involved in the selection of the myeloid vs. lymphoid lineage suggesting that C/EBPα primes lineage choice at the HSC level. Thus, our work highlight the complexities by which transcriptional circuitries are operating to maintain functional HSCs by balancing key cell fate options such as quiescence, cell death, self-renewal and differentiation.

## Materials and Methods

### Mouse Colony

Animals were housed according to institutional guidelines at the University of Copenhagen and housed according to institutional guidelines. Excision of the *Cebpa* allele was achieved by subjecting 10–12 weeks old *Cebpa* ^fl/fl^ or *Cebpa* ^fl/fl^;*Mx1Cre* mice to 3 injections (day 0, 2 and 4) with 200 ul polyinosinic-polycytidylic acid (pIpC) (1.5 mg/ml in PBS, GE Healthcare) as described previously [58]. After conditional deletion of *Cebpa*, the mice were treated with ciprofloxacin (100 mg/L, Actavis) in the drinking water. Mice were analysed 18–21 days after first pIpC injection.

### Ethics Statement

All animal work was done with approval from the Danish Animal Ethical Committee. This study was approved by the review board at the Faculty of Health Sciences, University of Copenhagen.

### Flow Cytometry

BM and PB was stained with antibodies and run on a LSRII (Becton Dickinson) or FACSAria (Becton Dickinson) and analysed using the FlowJo software. Student two-tailed T-test was used to test for significance.

### Transplantation Assays

All reconstitution assays were performed using the Ly-5 congenic mouse system. Briefly, whole BM or HSCs (LSK, CD150+, CD48−) from Ly-5.2 (CD45.2) donors were transplanted by tail vein injection into 10–12 week old lethally irradiated (900 cGy) Ly-5.1 (CD45.1) mice +/− Ly-5.1 support BM cells. The recipient mice were treated with ciprofloxacin (100 mg/L, Actavis) in the drinking water. For all transplantation assays, PB was analyzed at 3–4 weeks, 8 weeks and 16–18 weeks after transplantation. The BM from the recipients was analyzed at the experimental endpoint, i.e. 16–18 weeks after transplantation. Student two-tailed T-test was used to test for significance.

### Homing Assay

HSCs (LSK, CD150+, CD48−) were isolated as described above and stained with carboxyfluorescein diacetate succinimidyl ester (CFSE) (CellTrace, Invitrogen) according to manufacturer's protocol. Next, HSCs (3000/mouse) were resuspended in PBS+3% FCS and transplanted by tail vein injection into irradiated (900 cGy) Ly-5.1 (CD45.1) mice. Twelve hours later BM and spleen from recipients were isolated, enriched for c-Kit+ cells, and analyzed on a LSRII for CFSE+ cells. Student two-tailed T-test was used to test for significance.

### In Vitro Cell Culture

Single HSCs (LSK, CD150+, CD48−) were sorted directly to round-bottom 96-well plates and cultured in serum-free medium (StemSpan SFEM, StemCell Technologies) supplemented with 100 ng/ml SCF and 100 ng/ml TPO (Peprotech). Each well was examined by microscopy and cell numbers were recorded every other day for a week. P-values for differences in colony size were calculated using chi-square test.

### Continuous Live Imaging of HSCs

HSCs (LSK, CD150+, CD48−) were sorted and cultured in serum-free medium (StemSpan SFEM, StemCell Technologies) supplemented with SCF (100 ng/ml), IL-3 (10 ng/ml), IL-6 (10 ng/ml), TPO (100 ng/ml) (Peprotech), EPO (5 U/ml) (Promocell), penicillin (50 U), streptomycin (50 ug/ml) and Hepes (10 mM) (Gibco) in T12.5 flasks coated with fibronectin (Innovative Research). Using cell observer (Zeiss), images were acquired every 90 seconds using a VBA module controlling Zeiss AxioVision 4.8 software and analyzed using TTT software [59]. Student two-tailed T-test was used to test for significance.

### Gene Expression Profiling

RNA was purified from sorted HSCs (LSK, CD150+, CD48−), amplified, labeled and hybridized to the Mouse Gene 1.0 ST GeneChip Array (Affymetrix, Santa Clara, CA, USA). Raw gene expression data are available at the Gene Expression Omnibus (GEO) online database under ID GSE42498.

### ChIP-seq Analysis

ChIP-seq was performed using LSK or GMP cells from *Cebpa* ^fl/fl^ or *Cebpa* ^Δ/Δ^ mice. Chromatin from 100.000 or 500.000 cells was incubated with antibodies for histone marks (H3K4me3 and H3K27me3, Cell Signaling) or C/EBPα (Santa Cruz Biotechnology), respectively. The antibody-bound chromatin was captured with Protein-A sepharose beads, washed, de-cross-linked and precipitated. Precipitated DNA were mixed with 2 ng *E. Coli* DNA and amplified using NEB Next ChIP-seq sample prep reagent set 1 (New England Biolabs) according to manufacturer's protocol. Libraries were sequenced on an Illumina Genome Analyzer IIx or and Illumina Hiseq2000. Data was deposited in the NCBI Gene Expression Omnibus online database under ID GSE43007. See Table S10 for primers.

### Bioinformatic Analyses

All reads were mapped using bowtie 0.12.7 [60] using standard parameters (Figure S3B). C/EBPα peaks were called with MACS, v. 1.4 [61] with an IgG mock sample as control and using 1*10^−5^ as the p-value threshold. Clusters in both positional and regular heatmaps were identified by k-means clustering using the biopython Bio Cluster module [62]. In order to identify TF motifs in C/EBPα bound regions, sequences below the peak summit +/−70 bp were analyzed.

Additional and detailed Materials and Methods are available as Supporting Information (See Text S1).

## Supporting Information

Figure S1 *Cebpa* ^Δ/Δ^ mice have reduced cellularity within the BM and altered distribution of myeloid progenitors. (A) PCR on whole BM and spleen for wt, floxed (fl) and deleted (del) *Cebpa* allele from *Cebpa* ^fl/fl^ and *Cebpa* ^Δ/Δ^ mice. (B) Cytospins of whole BM from *Cebpa* ^fl/fl^ and *Cebpa* ^Δ/Δ^ mice. (C) BM cell number in femur and tibia of *Cebpa* ^fl/fl^ (n = 7) and *Cebpa* ^Δ/Δ^ (n = 7) mice. (D) Distribution of myeloid progenitors in *Cebpa* ^fl/fl^ and *Cebpa* ^Δ/Δ^ BM. (E) Mean of (D). *Cebpa* ^fl/fl^ (n = 6) and *Cebpa* ^Δ/Δ^ (n = 3). Data are represented as mean+SEM.
(EPS)

Figure S2 Gene expression analysis in *Cebpa* ^fl/fl^ and *Cebpa* ^Δ/Δ^ HSCs and ChIP-seq analysis of C/EBPα binding in LSK stem and progenitor cells. (A) Gene expression of selected genes in *Cebpa* ^fl/fl^ (n = 3) and *Cebpa* ^Δ/Δ^ (n = 3) HSCs was quantified using qPCR (B) Selected examples of ChIP-seq coverage of C/EBPα peaks in LSK cells (replicate 2). (C) Correlation of C/EBPα ChIP-seq replicates. (D) qPCR of selected genes in IgG and C/EBPα ChIP (replicate 2).
(EPS)

Figure S3 Analysis of replicate H3K4me3 and H3K4me27 samples. (A) Correlation of replicates in TSS +/−1000 bp for H3K4me3 and in gene bodies for H3K27me3. (B) Mapped reads of each of the ChIP-seq samples. (C) Selected examples of ChIP-seq coverage in LSK cells. (D) qPCR of selected genes (examples from Figure S3C) and genes which are part of both bivalent and stemness genes in IgG, H3K4me3 and H3K27me3 ChIP samples. For visualization purposes the H3K27me3 enrichment was normalized to IgG.
(EPS)

Figure S4 GO-term analysis of the bivalently marked cluster.
(EPS)

Figure S5 Analysis of H3K4me3 and H3K27me3 level in *Cebpa* ^fl/fl^ and *Cebpa* ^Δ/Δ^ in signatures identified in GSEA. The corresponding p-values were determined. The black line in the p-value histogram indicates P = 0.01.
(TIF)

Table S1 Gene expression analysis of *Cebpa* ^Δ/Δ^ HSCs and *Cebpa* ^fl/fl^ HSCs.
(XLS)

Table S2 Genes associated with C/EBPα bound regions in LSKs. C/EBPα peaks were associated to nearest TSS. Shown are the peak positions, the distance from the C/EBPα peaks to the nearest gene TSS, the gene expression fold changes between *Cebpa* ^fl/fl^ and *Cebpa* ^Δ/Δ^ HSCs and p-values for gene expression changes. NA designates genes not assigned on the microarray.
(XLSX)

Table S3 Motif analysis at C/EBPα bound regions in LSKs. Pos_hit and pos_no_hits designates the number of hits and the number with no hits in the tested dataset, respectively. Neg_hit and neg_no_hits designates the number of hits and the number with no hits in the background dataset, respectively.
(XLSX)

Table S4 Pathways, GO-terms and mouse phenotypes identified *via* GREAT analysis of C/EBPα bound regions in LSK cells.
(XLSX)

Table S5 Genes associated with C/EBPα bound regions in GMPs. C/EBPα peaks were associated to nearest TSS. Shown are the peak positions and the distance from the C/EBPα peaks to the nearest gene TSS.
(XLSX)

Table S6 De-regulated genes in *Cebpa* ^Δ/Δ^ HSCs and their association with nearest C/EBPα bound region. The TSSs of deregulated (>1.5 fold) genes were correlated to nearest C/EBPα bound region. Shown are gene expression (log2) in HSCs, fold change (FC), p-value and distance to nearest C/EBPα bound region.
(XLSX)

Table S7 Distance from the TSS of genes in cluster 6 to nearest C/EBPα bound region. Shown are normalized and scaled values for peak coverage at the TSS (+/−2000 bp) and in gene bodies (GB) in Cluster 6 for H3K4me3 and H3K27me3, gene expression (log2) in HSCs, fold change (FC), p-value and distance to nearest C/EBPα bound region.
(XLSX)

Table S8 GO-term analysis of bivalent genes in cluster 6. The significance of enriched GO terms was assessed using hypergeometric tests. In order to account for multiple testing, p-values were corrected by Bonferroni correction.
(XLSX)

Table S9 Genes constituting the stemness signature analyzed for gene expression in *Cebpa* ^Δ/Δ^ and *Cebpa* ^fl/fl^ HSCs and distance to nearest C/EBPα bound region.
(XLSX)

Table S10 Primer sets used for qPCR.
(XLSX)

Text S1 Detailed Materials and Methods.
(DOCX)
