# Supplementary figures and images for: C/EBPα Is Required for Long-Term Self-Renewal and Lineage Priming of Hematopoietic Stem Cells and for the Maintenance of Epigenetic Configurations in Multipotent Progenitors
Source: PLoS Genet. 2014 Jan 9;10(1):e1004079. doi: 10.1371/journal.pgen.1004079 (PMC3886906; doi:10.1371/journal.pgen.1004079)

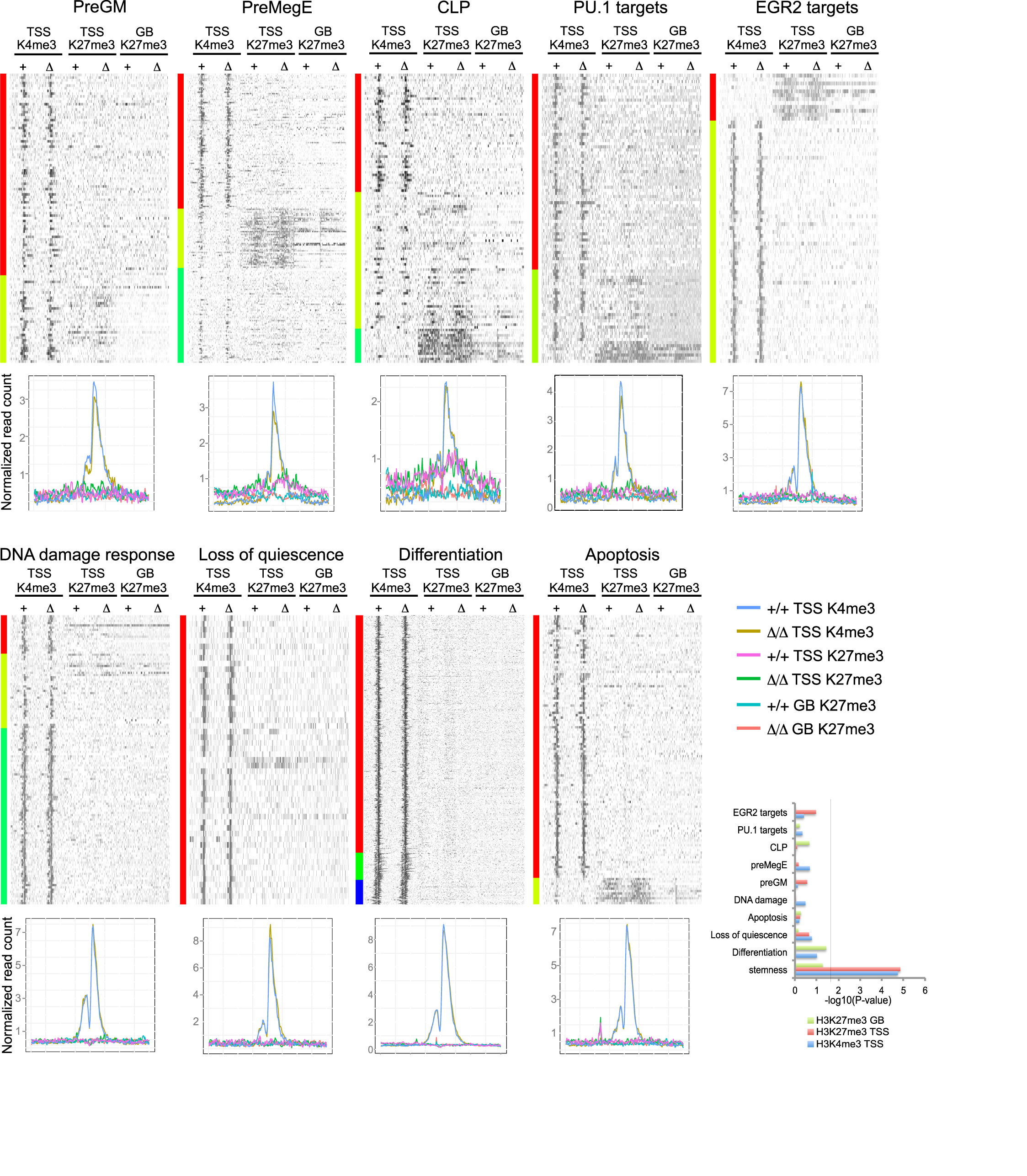

Supplement: Figure S5 — Analysis of H3K4me3 and H3K27me3 level in Cebpa fl/fl and Cebpa Δ/Δ in signatures identified in GSEA. The corresponding p-values were determined. The black line in the p-value histogram indicates P = 0.01. (TIF) [file pgen.1004079.s005.tif]
