# Supplementary material for: C/EBPα Is Required for Long-Term Self-Renewal and Lineage Priming of Hematopoietic Stem Cells and for the Maintenance of Epigenetic Configurations in Multipotent Progenitors
Source: PLoS Genet. 2014 Jan 9;10(1):e1004079. doi: 10.1371/journal.pgen.1004079 (PMC3886906; doi:10.1371/journal.pgen.1004079)
Supplement: Text S1 — Detailed Materials and Methods. (DOCX) [file pgen.1004079.s016.docx]

C/EBPα Is Required for Long-Term Self-Renewal and Lineage Priming of Hematopoietic Stem Cells and for the Maintenance of Epigenetic Configurations in Multipotent Progenitors

# Supporting Materials and Methods, Text S1

## Mouse Colony

Animals were maintained at the Department of Experimental Medicine at University of Copenhagen and housed according to institutional guidelines. All animal work was done with approval from the Danish Animal Ethical Committee. The *Mx1-Cre* mice have been described previously [[1](#_ENREF_1)]. The *Cebpa*^fl/fl^ line used in the present study was constructed by Lee et al. [[2](#_ENREF_2)]**.** Briefly, a *loxP* site was introduced 40 bp downstream of TSS in a *DraIII* site in the 5’ untranslated region. The PGK-*neo* gene with flanking *loxP* sites was introduced in the *EcoRI* site 2 kb downstream of the poly adenylation signal [[2](#_ENREF_2)]. All experimental animals had been backcrossed for at least 10 generations to the C57BL/6 background.

*Mx1-Cre* mice were genotyped by PCR using the primers GCCTGCATTACCGGTCGATGCAACGA and GTGGCAGATGGCGCGGCAACACCATT. *Cebpa*^fl/+^ mice were genotyped by PCR using the primers GACTCCATGGGGGAGTTAGAG and GCCTTGGAAAGTCACAGGAG. Excision of the *Cebpa* allele was achieved by subjecting 10-12 weeks old *Cebpa*^fl/fl^ or *Cebpa*^fl/fl^;*Mx1Cre* mice to 3 injections with polyinosinic-polycytidylic acid (pIpC) as described previously [[3](#_ENREF_3)]. Recombination was evaluated by competitive PCR using the primers GTCCTGCAGCCAGGCAGTGTCC, CCACTCACCGCCTTGGAAAGTCACA and CCGCGGCTCCACCTCGTAGAAGTCG, which gives rise to a 355 bp and 560 bp products for the deleted and floxed allele, respectively. After conditional deletion of *Cebpa,* the mice were treated with ciprofloxacin (100 mg/L, Actavis) in the drinking water.

## Flow Cytometry

For the analysis of HSCs (LSK, CD150+, CD48-), tibia and femurs were collected and crushed in PBS + 3% FCS, blocked with anti-CD16/32 and stained with antibodies against B220, CD3, CD11b, Gr1, Ter119, CD34, Sca-1, c-Kit (eBioscience), CD150 and CD48 (Biolegend) before being supplemented with 7AAD prior to analysis. For HSC (LSK, CD150+, CD48-) purification, c-Kit+ cells were enriched with anti-c-Kit beads by MACS (Miltenyi Biotec) prior to antibody staining. Red blood cells were removed by PharmLyse (Becton Dickinson) according to manufacturers protocol. For analysis purposes samples were run on a LSRII (Becton Dickinson), whereas FACS sortings were performed on a FACS Aria (Becton Dickinson). Analysis of hematopoietic progenitors was performed using antibodies against B220, CD3, CD11b, Gr1, Ter119, CD105, FcgRII/III, CD41, Sca-1, c-Kit (eBioscience) and CD150 (Biolegend) as described previously [[4](#_ENREF_4)]. For analysis of transplanted animals we included antibodies against CD45.1 (eBioscience) and CD45.2 (Becton Dickinson) in the analysis. For analysis of cell death the BM cells were stained for HSCs (LSK, CD150+, CD48-) with Annexin V and DAPI according to manufactures protocol (BD).

Peripheral blood (PB) was stained for B220, CD4, CD8a, CD11b, CD45.1 (e-Bioscience) and CD45.2 (Becton Dickinson) as described [[5](#_ENREF_5)]. The data was analyzed using the FlowJo software.

## Transplantation Assays

All reconstitution assays were performed using the Ly-5 congenic mouse system. For the non-competitive repopulation assay, one million unfractionated BM from *Cebpa*^fl/fl^ or *Cebpa*^Δ/Δ^ Ly-5.2 (CD45.2) were transplanted by tail vein injection into 10-12 week old lethally irradiated (900 cGy) Ly-5.1 (CD45.1) mice. For secondary transplantation, ½ femur equivalent was re-transplanted. The recipient mice were treated with ciprofloxacin (100 mg/L, Actavis) in the drinking water.

For whole BM competitive repopulation assays one million unfractionated BM cells from *Cebpa*^fl/fl^ or *Cebpa*^Δ/Δ^ Ly-5.2 (CD45.2) was mixed with one million competitor BM cells Ly-5.1 (CD45.1) and transplanted by tail vein injection into lethally irradiated (900 cGy) Ly-5.1 (CD45.1) mice. For secondary transplantations, one million whole BM cells were re-transplanted.

For HSC competitive repopulation assays, 20 freshly isolated HSCs (LSK, CD150+, CD48-, CD34-) from *Cebpa*^fl/fl^ or *Cebpa*^Δ/Δ^ Ly-5.2 (CD45.2) were mixed with 200.000 competitor BM cells Ly-5.1 (CD45.1) cells and transplanted by tail vein injection into irradiated (900 cGy) Ly-5.1 (CD45.1) mice. For secondary transplantations, one million whole BM cells were re-transplanted.

For all transplantation assays, PB was analyzed at 3-4 weeks, 8 weeks and 16-18 weeks after transplantation. The BM from the recipients was analyzed at the experimental endpoint, i.e. 16-18 weeks after transplantation.

## Homing Assay

HSCs (LSK, CD150+, CD48-) were isolated from *Cebpa*^fl/fl^ or *Cebpa*^Δ/Δ^ mice as described above and stained with carboxyfluorescein diacetate succinimidyl ester (CFSE) (CellTrace, Invitrogen) according to manufacturers protocol. Briefly, FACS sorted 10.000 HSCs were incubated at 37°C for 10 min with 10 μM CFSE in 200 μl PBS + 3% FCS. Next, the cells were washed three times as follows: 1 ml ice cold PBS + 3% FCS was added and cells were incubated 5 min on ice in the dark prior to centrifugation and removal of the supernatant. Finally, the HSCs (3000/ mouse) were resuspended in PBS + 3% FCS and transplanted by tail vein injection into irradiated (900 cGy) Ly-5.1 (CD45.1) mice. Twelve hours later BM and spleen from recipients were isolated, enriched for c-Kit+ cells as described above, and analyzed on a LSRII for CFSE+ cells.

## BrdU Analysis

*Cebpa*^fl/fl^ and *Cebpa*^Δ/Δ^ mice were intaperitoneally injected with BrdU (80 mg/kg mouse, Sigma) three hours prior harvest of the BM. BM cells were stained for HSCs (LSK, CD150+, CD48-) and BrdU according to manufactures protocol (Becton Dickinson).

## Ki67 Analysis

BM cells from *Cebpa*^fl/fl^ or *Cebpa*^Δ/Δ^ mice was stained for HSCs (LSK, CD150+, CD48-) and fixed in 4% PFA for 10 min at RT followed by permabilisation with 0,1% saponin in PBS + 3% FCS for 45 min at RT. Next, cells were stained with anti-Ki67 (Becton Dickinson) for 30 min at RT and with 0,5 μg/ml DAPI (Invitrogen) and analyzed on a LSRII (Becton Dickinson).

## DNA Damage Analysis

Whole BM from *Cebpa*^fl/fl^ or *Cebpa*^Δ/Δ^ mice was stained for HSCs (LSK, CD150+, CD48-) and fixed in 4% PFA for 10 min at RT followed by permabilisation with 0,1% saponin in PBS for 45 min at RT. Next, cells were stained with anti-γH2A.X (Milipore) for 60 min at RT, washed and stained with goat anti-mouse alexa fluor 594 (Invitrogen) at RT for 30 min and subjected to FACS analysis on a LSRII (Becton Dickinson).

## Gene Expression Profiling

HSCs (LSK, CD150+, CD48-) were sorted directly into RLT buffer and RNA was purified according to manufacturers protocol (RNeasy micro kit, Qiagen). RNA was amplified using the Pico amplification kit (Nugen Technologies), according to manufacturers instructions. Subsequently, 2.5ug of amplified cDNA was fragmented and labeled using Encore Biotin Module V2 according to manufacturers instructions (Nugen Technologies). The labeled samples were hybridized to the Mouse Gene 1.0 ST GeneChip Array (Affymetrix, Santa Clara, CA, USA). The array was scanned with GeneArray 3000 and CEL files generated in GeneChip Command Console Software (AGCC) (Affymetrix). The data were modelled using the Robust Multichip Average approach followed by mean one step Probe Set summarization using Partek Genomics Suite 6.5 software. Raw gene expression data are available at the Gene Expression Omnibus (GEO) online database under ID GSE42498.

RNA expression levels were quantified by qRT-PCR (Roche Lightcycler 480) and normalized to beta-actin. All primer sets are listed in Table S10.

## Gene Set Enrichment Analysis (GSEA)

Gene Set Enrichment Analysis was performed using the GSEA v3.0 (http://www.broadinstitute.org/gsea/). Gene sets originated from the MSigDB ([www.broadinstitute.org/gsea/msigdb](http://www.broadinstitute.org/gsea/msigdb)) and from a list of curated signatures extracted from [[6](#_ENREF_6),[7](#_ENREF_7),[8](#_ENREF_8)]. The curated signatures were kindly provided by Susan Moore and Claus Nerlov. The Signature for PU.1 target genes was generated from [[9](#_ENREF_9)] and defined as genes being upregulated ≥ 2, p<0,05 in PU.1 expressing cells (*Sfpi1*^BN/BN^) vs. PU.1 knockout cells (*Sfpi1*^-/-^).

## ChIP-seq Analysis

LSK or GMP cells from *Cebpa*^fl/fl^ or *Cebpa*^Δ/Δ^ mice were FACS sorted and cross-linked for 10 min at RT in 1% formaldehyde and quenched with 0,125 M glycine for 2 min. Cells were washed twice in cold PBS, resuspended in lysis buffer (100mM NaCl, 66mM tris-HCl, 5mM EDTA, 0.3% SDS, 1.5% triton X-100) and incubated for 20 min at RT with vortexing every 5 min. Cell lysates were sonicated (30 cycles, 15 s burst, 30 s break) using a bioruptor (Diagenode), centrifuged for 10 min and supernatants were frozen in liquid nitrogen and stored at -80°C. Chromatin from 100.000 or 500.000 cells was pre-cleared with Protein-A sepharose beads and incubated with antibodies for histone marks (H3K4me3 and H3K27me3, Cell Signaling) or C/EBPα (Santa Cruz Biotechnology) for 12 h at 4°C with rotation, respectively. The antibody-bound chromatin was captured with Protein-A sepharose beads (3 h at 4°C with rotation) and beads were rinsed twice with RIPA buffer (140mM NaCl, 10mM Tris-HCl, 1mM EDTA, 1% Triton-X100, 0.1% SDS, 0,1% sodium deoxycholate, 1mM PMSF) (1 ml RIPA, 1000 g, 2 min), followed by extensive washing cycles (1 ml wash buffer, incubate with rotation 5 min, spin for 2 min at 1000g) using RIPA (2x for C/EBPα ChIPs and 3x for histone mark ChIPs), RIPA with 0.5 M NaCl (2x for C/EBPα ChIPs and 1x for histone mark ChIPs), LiCl buffer (250mM LiCl, 10mM Tris-HCl, 1mM EDTA, 0.5% NP-40, 0,1% sodium deoxycholate) (1x for all ChIPs) and TE (10mM Tris-HCl, 1mM EDTA) (2x for all ChIPs). The complexes were treated with RNase A for 30 min at 37°C followed by addition of SDS (0,5%) and proteinase K (0,5 mg/ml) and an additional incubation for 16 hours at 37°C . Finally, chromatin complexes were de-cross-linked by incubation at 65°C for 6 h, phenol-chloroform extracted and precipitated using NaOAc and EtOH supplemented with glycogen. Precipitated DNA were mixed with 2 ng *E. Coli* DNA and amplified using NEBNext ChIP-seq sample prep reagent set 1 (New England Biolabs) according to manufacturers protocol. Libraries were sequenced on an Illumina Genome Analyzer IIx or and Illumina Hiseq2000. Enrichment was validated by qPCR (Roche Lightcycler 480) using ratios of a positive detector primer set versus a negative (*Mef2* or *Sfi2*). All primer sets are listed in Table S10.

## ChIP-seq Data Analysis

All reads were mapped using bowtie 0.12.7 [[10](#_ENREF_10)] using standard parameters (Figure S3B). C/EBPα peaks were called with MACS, v. 1.4 [[11](#_ENREF_11)] with an IgG mock sample as control and using 1*10^-5^ as the p-value threshold, generating approximately 18,000 candidate regions. Peak height was calculated in each sample and a minimum peak height was set, dependent on the background in the individual samples. Peaks present in both C/EBPα samples were extracted and regions with high IgG levels were filtered out. In addition, clustering analysis was used to remove artifacts and sticky genomic regions. All peaks were manually inspected. GMP peaks were called using MACS2 (<https://github.com/taoliu/MACS/downloads>) with the following specifications: macs2 callpeak –t GMP.tagAlign.gz -c IgG_mock.tagAlign.gz -f BED -n GMP_vs_IgG -g mm -p 1e-3 --to-large. Overlapping peaks were subjected to filtration based on the p-value given by MACS2 (min 10^-14^), and by peak height when comparing with the individual global sample background (peak height should be greater than the background height of the 0.01% highest random regions). Regions with high IgG levels were filtered out. For all samples, raw genome coverage was converted to the BigWig format for visualization in the UCSC genome browser [[12](#_ENREF_12)], Genes were defined using the mm9 RefSeq set, taking the longest supported isoform for genes with multiple isoforms. The ChIP-seqs were performed as biological replicates and the correlation coefficients, r^2^, were calculated (Figure S2C, Figure S3A). For positional coverage heatmaps and plots, read coverage was calculated in both 200 40 bp windows surrounding the TSS, and over whole genes split into 200 windows of equal size. The coverage was normalized to sequencing depth, to the length of the region (TSS=8000 bp, gene body=gene length), and followed by quantile normalization [[13](#_ENREF_13)]. For normal heatmaps, the coverage in TSS for both H3K4me3 and H3K27me3 was calculated as the normalized coverage in TSS +/- 2000 bp. The gene body coverage was calculated as coverage in the entire gene normalized to gene length. Samples were scaled for visual purposes. For testing for significant differences between bound H3K4me3 levels or H3K27me3 levels in TSSs or across gene bodies, we summed up the total coverage in the windows described above, and employed the Wilcoxon rank-sum test.

Distances to C/EBPα peaks were calculated from the peak center to the TSS. Clusters in both the positional and the regular heatmap were found by k-means clustering using the biopython  Bio.Cluster module [[14](#_ENREF_14)]. The Euclidian distance was used to compute the distance matrix between the regions and 10^5^ passes of the clustering algorithm were performed.  Gene expression and C/EBPα bound regions were not included in the clustering, but added in the correct order afterwards. To facilitate co-vizualization, chip-seq and microarray data were scaled by subtracting the mean and dividing by the standard deviation. In order to identify transcription factor motifs below in C/EBPα bound regions, Jaspar [[15](#_ENREF_15)] and Transfac [[16](#_ENREF_16)] motifs were fitted to a log normal background normalization based on sequence from 2000 bp upstream of all mm9 RefSeq genes. Sequences below the peak summit +/- 70 bp were scored against these motifs using the Bioconductor package PWMEnrich (Robert Stojnic (2012). PWMEnrich: PWM enrichment analysis. R package version 1.2.0.), generating affinity scores (average odds score over the whole sequence), as well as p-values (hypergeometric tests against hits in the background set).

TET 1 bound regions (FDR = 0.1) were extracted from [[17](#_ENREF_17)] and their overlap with cluster 6 genes was determined. To test for significance, we employed the hypergeometric test using 1000 random samples of all RefSeq gene symbols as background. To test for significance of H3K4me3 levels between from *Cebpa*^fl/fl^ or *Cebpa*^Δ/Δ^ mice we employed the Wilcoxon rank-sum test. Data was deposited in the NCBI Gene Expression Omnibus online database under ID GSE43007.

## Gene Ontology (GO) Analysis

Gene Ontology (GO) analysis was performed with hypergeometric tests using the ClueGo plugin [[18](#_ENREF_18)] for Cytoscape [[19](#_ENREF_19)]. GO consists of a tree of biological terms, where features, genes, are inherited from child node to parent node. Thus, in order to better meet the criteria of independence given by the hypergeometric test, only terms from one general level was used for testing. GO level was set to three and tests were performed against the Biological Process subtree, where GO term fusion was allowed for redundancy reduction. In order to account for multiple testing, p-values were corrected by Bonferroni correction.

# Supporting References

1. Kuhn R, Schwenk F, Aguet M, Rajewsky K (1995) Inducible gene targeting in mice. Science 269: 1427-1429.

2. Lee YH, Sauer B, Johnson PF, Gonzalez FJ (1997) Disruption of the c/ebp alpha gene in adult mouse liver. Molecular and cellular biology 17: 6014-6022.

3. Weischenfeldt J, Damgaard I, Bryder D, Theilgaard-Monch K, Thoren LA, et al. (2008) NMD is essential for hematopoietic stem and progenitor cells and for eliminating by-products of programmed DNA rearrangements. Genes Dev 22: 1381-1396.

4. Hasemann MS, Schuster MB, Frank AK, Theilgaard-Monch K, Pedersen TA, et al. (2012) Phosphorylation of serine 248 of C/EBPalpha is dispensable for myelopoiesis but its disruption leads to a low penetrant myeloid disorder with long latency. PLoS One 7: e38841.

5. Thoren LA, Liuba K, Bryder D, Nygren JM, Jensen CT, et al. (2008) Kit regulates maintenance of quiescent hematopoietic stem cells. J Immunol 180: 2045-2053.

6. Venezia TA, Merchant AA, Ramos CA, Whitehouse NL, Young AS, et al. (2004) Molecular signatures of proliferation and quiescence in hematopoietic stem cells. PLoS biology 2: e301.

7. Mansson R, Hultquist A, Luc S, Yang L, Anderson K, et al. (2007) Molecular evidence for hierarchical transcriptional lineage priming in fetal and adult stem cells and multipotent progenitors. Immunity 26: 407-419.

8. Pronk CJ, Rossi DJ, Mansson R, Attema JL, Norddahl GL, et al. (2007) Elucidation of the phenotypic, functional, and molecular topography of a myeloerythroid progenitor cell hierarchy. Cell Stem Cell 1: 428-442.

9. Kamath MB, Houston IB, Janovski AJ, Zhu X, Gowrisankar S, et al. (2008) Dose-dependent repression of T-cell and natural killer cell genes by PU.1 enforces myeloid and B-cell identity. Leukemia 22: 1214-1225.

10. Langmead B, Trapnell C, Pop M, Salzberg SL (2009) Ultrafast and memory-efficient alignment of short DNA sequences to the human genome. Genome biology 10: R25.

11. Zhang Y, Liu T, Meyer CA, Eeckhoute J, Johnson DS, et al. (2008) Model-based analysis of ChIP-Seq (MACS). Genome biology 9: R137.

12. Kent WJ, Sugnet CW, Furey TS, Roskin KM, Pringle TH, et al. (2002) The human genome browser at UCSC. Genome research 12: 996-1006.

13. Bolstad BM, Irizarry RA, Astrand M, Speed TP (2003) A comparison of normalization methods for high density oligonucleotide array data based on variance and bias. Bioinformatics 19: 185-193.

14. de Hoon MJ, Imoto S, Nolan J, Miyano S (2004) Open source clustering software. Bioinformatics 20: 1453-1454.

15. Portales-Casamar E, Thongjuea S, Kwon AT, Arenillas D, Zhao X, et al. (2010) JASPAR 2010: the greatly expanded open-access database of transcription factor binding profiles. Nucleic acids research 38: D105-110.

16. Matys V, Kel-Margoulis OV, Fricke E, Liebich I, Land S, et al. (2006) TRANSFAC and its module TRANSCompel: transcriptional gene regulation in eukaryotes. Nucleic acids research 34: D108-110.

17. Williams K, Christensen J, Pedersen MT, Johansen JV, Cloos PA, et al. (2011) TET1 and hydroxymethylcytosine in transcription and DNA methylation fidelity. Nature 473: 343-348.

18. Bindea G, Mlecnik B, Hackl H, Charoentong P, Tosolini M, et al. (2009) ClueGO: a Cytoscape plug-in to decipher functionally grouped gene ontology and pathway annotation networks. Bioinformatics 25: 1091-1093.

19. Smoot ME, Ono K, Ruscheinski J, Wang PL, Ideker T (2011) Cytoscape 2.8: new features for data integration and network visualization. Bioinformatics 27: 431-432.
